# Serological and Molecular Evidence of Q Fever in Domestic Ruminants in Bangladesh

**DOI:** 10.1155/2016/9098416

**Published:** 2016-04-30

**Authors:** Md. Arifur Rahman, Md. Mahbub Alam, Md. Aminul Islam, A. K. Fazlul Haque Bhuiyan, A. K. M. Anisur Rahman

**Affiliations:** ^1^Department of Medicine, Faculty of Veterinary Science, Bangladesh Agricultural University (BAU), Mymensingh 2202, Bangladesh; ^2^Department of Animal Breeding and Genetics, Faculty of Animal Husbandry, BAU, Mymensingh 2202, Bangladesh

## Abstract

The objective of this study was to know the herd and animal level prevalence of Q fever in domestic ruminants in some selected districts in Bangladesh. Randomly collected 111 bulk milk and 94 sera samples of cattle, sheep, and goats were tested by indirect ELISA (iELISA). DNA extracted from 23 aborted fetal membranes was analyzed by real time (rt) PCR. The positive cut-off value of iELISA in bulk milk and individual animal sera was ≥30% and ≥40%, respectively. The overall herd level prevalence of Q fever in dairy cattle was 15.6%. The prevalence of Q fever in dairy cattle was significantly higher in Sirajganj in comparison to Satkhira District (*P* < 0.01). The overall seroprevalence of Q fever in domestic ruminants was 5.06%. Although statistically insignificant, the seroprevalence of Q fever was relatively higher in sheep (9.52%) in comparison to goats (3.33%) and cattle (3.57%). Out of 23 aborted fetal membranes, only one sheep placenta was positive in rt PCR. Q fever is present in all of the three important species of domestic ruminants in Bangladesh. It may have some role in sheep abortion as the seroprevalence is relatively higher and also one sheep placenta is rt PCR positive.

## 1. Introduction

Q (for Query) fever is a ubiquitous zoonosis caused by an obligate intracellular bacterium* Coxiella* (*C.*)* burnetii*. It has been reported from all over the world except Antarctica and possibly New Zealand [1, 2]. The primary reservoirs of* C. burnetii* are cattle, sheep, and goats. However, the infection has been reported in other mammals (humans, cats, dogs, rodents, rabbits, horses, swine, camels, water buffalo, and marine mammals), ticks and other arthropods, birds, fish, and reptiles [3, 4]. The common manifestations of Q fever in ruminants are abortion, stillbirth, premature delivery, and delivery of weak offspring [2]. Indeed these clinical manifestations are usually observed in sheep and goats and Q fever is mostly asymptomatic in cattle. Clinically infected cows may develop infertility, metritis, and mastitis [5].

In humans, Q fever is mostly asymptomatic but may be responsible for acute or chronic disease conditions such as influenza-like illness, pneumonia, hepatitis, meningoencephalitis, myocarditis, endocarditis, and chronic fatigue syndrome in persistently infected patients and may contribute to abortion and stillbirth in pregnant women [2, 6].

Diagnosis of Q fever in animals is based on detection of bacteria, bacterial DNA, or antibodies [7]. Although these bacteria can grow in axenic (host cell-free) media, isolation is time consuming and hazardous for the laboratory workers [8]. In addition, Q fever isolation techniques require a Biosafety Level 3 Laboratory (BSL-3). Mostly,* C. burnetii* exposure in animals can be screened indirectly by serological tests. The CFT (OIE recommended test) and ELISA (EU recommended test) are the two most commonly used serological tests in this purpose. However, CFT protocol is complex and fails to detect antibodies in sheep or goats [9]. The ELISA is reported to be highly sensitive and specific for the diagnosis of Q fever [10]. Moreover, ELISA can be used to detect antibodies in bulk milk and individual animal serum. The bacterial DNA can be detected by using PCR [11].

Although Q fever is present worldwide, its status in animals, humans, arthropods, birds, wild animals, and other reservoirs in Bangladesh is not known except one report on serological evidence in cattle and goats [12]. Nevertheless, the reproductive diseases in dairy cattle [13–15] are endemic in Bangladesh. So the objectives of this paper are to determine the herd level prevalence of Q fever in dairy cattle and goats, to estimate the animal level prevalence of Q fever in cattle, sheep, and goats originated from herds having previous history of abortion, and to detect* C. burnetii* DNA from aborted fetal membranes of cattle, goat, and sheep.

## 2. Materials and Methods

### 2.1. Milk Samples

**Figure 1:**
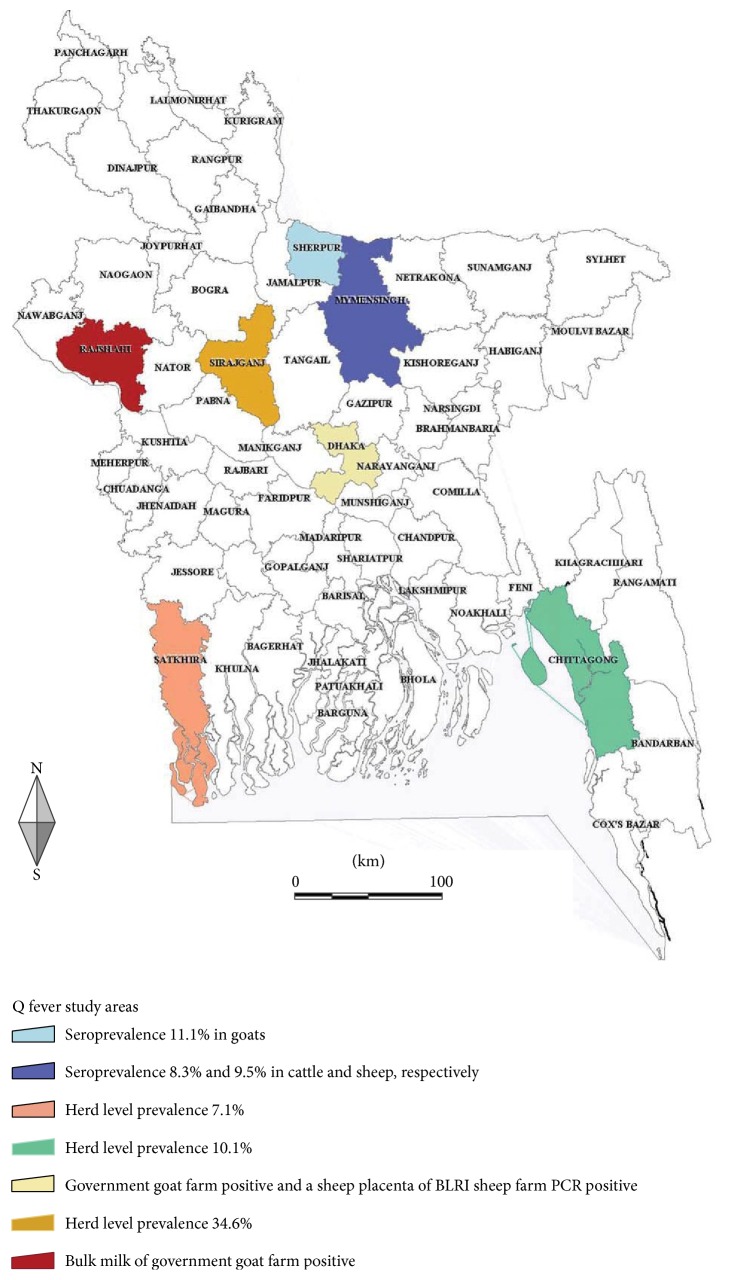


This study used milk samples from two previous studies, which were undertaken in the Department of Medicine, BAU, Mymensingh 2202. In one study, 399 randomly collected bulk milk samples were examined for somatic cell count from where 94 samples were used in this study. The history of reproductive failure in the selected dairy herds was not known. In another study, 17 milk ring test positive samples were sent to Belgium for isolation of* Brucella* spp., which were also used for this study. The districts of Bangladesh included in this study are shown in Figure 1.

### 2.2. Serum Sample Collection

Serum samples were collected from a serum bank in the Department of Medicine, BAU, Mymensingh. Those samples were randomly collected to study brucellosis in cattle, sheep, and goats in different districts of Bangladesh in 2007 and 2008 [16]. Ninety-four (94) serum samples were collected from 40 herds of the Mymensingh and Sherpur Districts out of 58 having some abortion (known from the owners) in the last year.

### 2.3. DNA Samples of Placentas

Twenty-three DNA samples (5 from cattle, 10 from goats, and 8 from sheep) extracted from aborted fetal membranes for the detection of* Brucella* spp. were also used in this study. DNA was extracted using the DNeasy spin column kit (QIAGEN) according to the manufacturer's protocol.

### 2.4. Herd and Animal Level Data Collection

Animal level data on age, breed, sex, and pregnancy status and herd level data on herd size, herd composition, and location of the herd were collected from available database of serum samples.

For milk samples, the location of the farm and number of lactating cows in herd were collected from the bovine mastitis database.

### 2.5. Indirect ELISA Test

#### 2.5.1. Preparation of Milk and Sera Samples

The milk and sera samples were prepared according to the instructions of commercial kit. In brief, 10 milliliters of milk from each selected herd was collected for testing antibody against* C. burnetii* exposure. The samples were centrifuged and the nonfat fraction was stored at −20°C until tested for antibodies against* C. burnetii*. Before testing, herd milk samples were prepared at 1 : 5 dilution using diluted (1 : 10) wash solution. Sera of the selected animals were removed from the serum bank and prepared at 1 : 400 dilution by using diluted wash solution.

#### 2.5.2. Test Procedure

All reagents were taken into 18–26°C before use. The reagents were mixed by shaking gently. All samples were tested in duplicate and the optical densities (OD) of the samples were averaged and corrected by subtracting the OD of the negative control. Both milk and serum based tests were performed using the commercial CHEKIT Q Fever Antibody ELISA Test Kit (IDEXX, Liebefeld-Bern, Switzerland) based on* C. burnetii* inactivated phase 1 and phase 2 antigens [10]. The positive cut-off value (S/P ratio) of iELISA in bulk milk and individual animal sera was ≥30% and ≥40%, respectively.

### 2.6. Real Time PCR

The real time (rt) PCR assay was performed using a 7500 rt PCR System (Applied Biosystems). Samples were considered positive with a cycle threshold (Ct) < 40 [17]. It was performed in Veterinary Agrochemical Research Centre (CODA-CERVA) in Brussels, Belgium.

### 2.7. Statistical Analysis

The association of herd and animal level factors with Q fever prevalence was analyzed by *χ* ^2^ test using R 3.1.0 (The R Foundation for Statistical Computing 2014).

## 3. Results

### 3.1. Descriptive Statistics

The serum samples were collected from 40 herds where there was history of abortion in any of the three domestic ruminant species in the last year. The herd size varied from 1 to 20 with a median of 3 animals. Thirteen herds consisted of only cattle, 13 of only goats, 8 of both cattle and goats, and 6 of only sheep. In 55.0% (22/40) herd's aborted materials were disposed by burial but in the rest of the herds the materials were thrown away in the field or in nearby water bodies. About 35% (14/40) farmers were found to keep sheep (7.5%) or goats (27.5%) inside their house at night.

The age of cattle varied from 4 months to 12 years with a median of 6 years. The range and median age of goats and sheep, respectively, were 2.5 months to 4 years and 2 years, 1 month to 4 years and 8 months. Among cattle 82.0% were female and indigenous and all of the sheep were indigenous and 74.2% of them were female but 80.0% and 94.0% goats were female and Black Bengal breed type, respectively. The range of positive S/P value in cattle herds was 41.4 to 123.0.

### 3.2. Herd Level Prevalence of Q Fever in Dairy Cattle

A summary of ELISA test results on the presence of* C. burnetii* antibodies in herd milk is presented in Table 1. An overall herd level prevalence of Q fever in dairy cattle was 15.6% (95% Confidence Interval (CI): 9.4–23.8) (Table 1). The distribution of Q fever in dairy herds is shown in Table 2. The prevalence of Q fever was significantly higher in Sirajganj (34.6%) in comparison to Satkhira District (*P* < 0.01). Although statistically insignificant, the prevalence of Q fever was relatively higher in herds having only Friesian cross (13.7%) and both Sahiwal and Friesian breed together (27.8%) in comparison to Sahiwal cross.

### 3.3. Seroprevalence of Q Fever in Cattle, Goats, and Sheep

The summary of ELISA test results on the presence of* C. burnetii* antibodies in serum samples is provided in Table 3. Out of 94 sera samples tested, the ages of 15 (10 sheep and 5 goats) animals were below six months (two seropositive sheep), which were excluded from the result in estimating seroprevalence (maternal immunity). The overall seroprevalence of Q fever in domestic ruminants was 5.06% (95% CI: 1.63–13.14). Three point seven nine percent (3.79%) sera samples were Q fever suspect and 91.13% were Q fever negative. The range of positive S/P value was 42.70 to 49.80%. The distribution of Q fever seroprevalence in domestic ruminants is shown in Table 4. The seroprevalence of Q fever was found to be higher in sheep (9.52%, 95% CI: 1.67–31.83) in comparison to goat (3.33%, 95% CI: 0.17–19.05) and cattle (3.57%, 95% CI: 0.18–20.24) but it was statistically insignificant. The seroprevalence of Q fever varied according to sex, pregnancy status, and study areas but none was significant statistically.

The demographic characteristics of the four Q fever seropositive domestic ruminants are shown in Table 5. Both seropositive sheep were from the same location (Unions/Sub-Upazila of Mymensingh Sadar Upazila/subdistrict).

### 3.4. Real Time PCR Result

*Coxiella burnetii* DNA was detected from only one sheep placenta. The remaining 22 samples were negative.

## 4. Discussion

In this study the herd level prevalence of Q fever in cattle based on bulk milk and animal level seroprevalence of Q fever in cattle, goats, and sheep were estimated by using indirect ELISA test. The overall prevalence of Q fever in bulk cow milk was 15.6% indicating that Q fever is an existing disease in dairy cattle population in Bangladesh. The herds under study were originated from major milk pockets of Bangladesh like Sirajganj, Chittagong, and Satkhira Districts (Figure 1). The sample size was very small and the sample does not represent the dairy herds of Bangladesh. It was also a limitation of this study. Due to the lack of fund it was not possible to include more samples in this study. So the herd level prevalence of Q fever we obtained may not represent the true status of this disease in dairy herds of the study areas. A widely variable and much higher herd level prevalence of Q fever (57.8 to 78.6%) was reported from different corners of the world [18–21]. Dairy cattle are usually chronically infected with Q fever and shed* C. burnetii* in the milk [22]. It is also stated that chronically infected dairy cattle are the most important source of human infection [1]. Another important source of human infection is the manipulation of fetus and its fluids and placentas from aborted small ruminants without safety protection measures. As Q fever is a zoonosis and it exists in animals of Bangladesh it is also supposed to be present in humans. Due to lack of reporting, awareness, and nonspecific influenza-like symptoms of this disease in humans, it may be overlooked and remained undiagnosed in human diagnostic laboratories. Due to lack of reporting from animals, the physicians are also unaware about this disease in humans. As a result, physicians usually do not refer flu-like cases for Q fever diagnosis. Both in humans and in animals, inhalation of bacteria present in the environment is the main route of infection. So dairy workers, animal caretakers, and pyrexia of unknown origin cases should be regularly tested for Q fever. Moreover, consumption of contaminated raw milk may produce infection in humans [1]. Indeed, the Bangladeshi population seldom ingests the raw milk.

We have tested only bulk milk, which does not allow identification of individual cows infected with Q fever. However, it is very useful for screening herds under disease surveillance system. A large epidemiologic study including representative dairy herds of Bangladesh will help to reveal the herd level status of this disease in Bangladesh. Out of three study areas, significantly higher prevalence of Q fever was found in dairy herds of Sirajganj than Satkhira District. The cattle management system in Sirajganj area slightly varies from that of other parts of Bangladesh. In the dry season, the cattle graze freely and remain in the pasture (“Bathan”) for almost six months (December to May). As a result, a lot of intermingling among cattle of different owners occurs during that period. Intermixing of cattle from different owners may facilitate the transmission of infection in dairy cattle herds of this area. In some herd, presence of sheep is also noticed in that period. Environment conditions in dry season could play a role in the survival of the bacteria and facilitate the transmission between animals as well. Similarly, higher prevalence of Q fever in loose housing system was also reported by Paul et al. [10]. Capuano et al. [23] also reported relatively higher seroprevalence of Q fever in herds housed in winter but turned out in spring than those housed permanently. Like other infectious diseases, Q fever was reported to be significantly associated with increased herd size [23, 24]. In this study, the prevalence of Q fever in contrast was a bit higher in smaller herds. However, the difference was not significant statistically. The prevalence of Q fever was relatively higher in herds having Friesian cross and in herd containing both Sahiwal and Friesian breed together although the difference was not significant statistically. Other authors had also reported significantly higher level of Q fever prevalence in Holstein breed [10, 23].

We have observed relatively higher seroprevalence of Q fever in sheep than cattle and goats. Similar observations were also reported by other authors [25, 26]. The prevalence of Q fever was reported to be significantly higher with the age of the animals [27–29]. We have also observed that the age of the seropositive animals is ≥10 months.

In our study, serum samples of the animals were originated from herds where there was history of abortion in previous year. Out of four seropositive cases two were in sheep indicating that Q fever might have some role in sheep abortion. Our rt PCR result also supports this hypothesis. An rt PCR Q fever positive result in the placenta means a contact with the bacteria. To confirm an abortion caused by* Coxiella burnetii* is necessary to detect histopathology lesions in the aborted fetus and placenta. Significantly higher seroprevalence of Q fever in sheep had also been reported by Berri et al. [30]. The immunosuppressive effects of pregnancy may be responsible for the increased multiplication of the organism in the placenta and thereby the higher seroprevalence [31].

It is revealed from this study that Q fever is present in all of the three important domestic ruminant species in Bangladesh. It may have some role in sheep abortion as the seroprevalence is relatively higher and one sheep placenta is rt PCR positive.

## Figures and Tables

**Table 1:** Summary of iELISA tests results on the presence of *Coxiella burnetii* antibodies (S/P values) in milk samples.

Test result	Number of herds/flocks	Apparent prevalence	95% CI	Range of S/P values (%)	Mean S/P values (%)
Positive (S/P ≥ 30%)	17 (cattle)	15.6%	9.4–23.8	41.4–123.0	81.3
Negative (S/P < 30%)	92	84.4%	76.2–90.6	0–25.9	5.6
Positive (S/P ≥ 30%)	2 (goats)^*∗*^			421.6 and 424.2	

^*∗*^Both goat flocks were positive; CI: Confidence Interval.

**Table 2:** Distribution of herd level prevalence of Q fever based on iELISA using bulk milk.

Variable	Tested	Positive (>40%)	Prevalence	95% CI	*χ* ^2^ test *P* value
District					<0.01
Satkhira	28	2	7.1	0.9–23.5	
Chittagong	55	6	10.9	4.1–22.2	
Sirajganj	26	9	34.6	17.2–55.6	
Number of lactating cows					1
>5	22	3	13.6	2.9–34.9	
1 to 5	87	14	16.1	9.1–15.5	
Breed composition					0.29
Sahiwal cross	18	2	11.1	1.4–34.7	
Friesian cross	73	10	13.7	6.8–23.8	
Both	18	5	27.8	9.7–53.5	

The other two bulk milk samples were collected from two government goat farms in Savar, Dhaka, and Rajshahi Districts; CI: Confidence Interval.

**Table 3:** Summary of iELISA tests results on the presence of *Coxiella burnetii* antibodies (S/P values) in serum samples.

Test result	Number	Prevalence (%)	95% Confidence Interval	Range of S/P values (%)	Mean S/P values (%)
Positive (S/P ≥ 40%)	4	5.06	1.63–13.14	42.70–49.80	45.35
Suspect (30% ≤ S/P < 40%)	3	3.79	0.98–11.45	30.10–34.50	32.40
Negative (S/P < 30%)	72	91.13	82.04–96.06	0–29.80	5.13

**Table 4:** The distribution of seroprevalence of Q fever in domestic ruminants.

Variable	Tested	Positive	Prevalence (95% CI)	*χ* ^2^ test *P* value
Species				0.55
Cattle	28	1	3.57 (0.18–20.24)	
Sheep	21	2	9.52 (1.67–31.83)	
Goats	30	1	3.33 (0.17–19.05)	
Sex				1.00
Male	15	1	6.67 (0.34–33.96)	
Female	64	3	4.69 (1.22–13.96)	
Pregnancy				0.63
No	38	1	2.63 (0.14–15.43)	
Yes	26	2	7.69 (1.34–26.59)	
Male	15	1	6.67 (0.34–33.96)	
District				1.00
Sherpur	25	1	4.0 (0.21–22.32)	
Cattle	16	0	0 (0–24.07^*∗*^)	
Sheep	0	0		
Goats	9	1	11.11 (0.58–49.33)	
Mymensingh	54	3	5.56 (1.44–16.34)	
Cattle	12	1	8.33 (0.44–40.25)	
Sheep	21	2	9.52 (1.67–31.83)	
Goats	21	0	0 (0–19.24^*∗*^)	

CI: Confidence Interval; ^*∗*^97.5% Confidence Interval.

**Table 5:** Characteristics of the four Q fever seropositive domestic ruminants.

Farmer ID	Area	Species	Age	Breed	Sex	Body weight	S/P value (%)
Fa 50	Sirta, Mymensingh Sadar	Cattle	6 years	Indigenous	Female	200	42.7
Fa 268	Noyabil, Sherpur	Goat	1 year	Black Bengal	Female	6	49.8
Fa 543	Buror Chor, Mymensingh Sadar	Sheep	10 months	Indigenous	Male entire	12	43.4
Fa 548	Buror Chor, Mymensingh Sadar	Sheep	1.5 years	Indigenous	Female	18	45.5

## References

[1] Maurin M., Raoult D. (1999). Q fever. *Clinical Microbiology Reviews*.

[2] Angelakis E., Raoult D. (2010). Q fever. *Veterinary Microbiology*.

[3] Babudieri B. (1959). Q fever: a zoonosis. *Advances in Veterinary Science*.

[4] Porter S. R., Czaplicki G., Mainil J., Guattéo R., Saegerman C. (2011). Q fever: current state of knowledge and perspectives of research of a neglected zoonosis. *International Journal of Microbiology*.

[5] To H., Htwe K. K., Kako N. (1998). Prevalence of *Coxiella burnetii* infection in dairy cattle with reproductive disorders. *Journal of Veterinary Medical Science*.

[6] Wildman M. J., Smith E. G., Groves J., Beattie J. M., Caul E. O., Ayres J. G. (2002). Chronic fatigue following infection by *Coxiella burnetii* (Q fever): ten-year follow-up of the 1989 UK outbreak cohort. *Quarterly Journal of Medicine*.

[7] Rodolakis A. (2006). Q fever, state of art: epidemiology, diagnosis and prophylaxis. *Small Ruminant Research*.

[8] Omsland A., Hackstadt T., Heinzen R. A. (2013). Bringing culture to the uncultured: *Coxiella burnetii* and lessons for obligate intracellular bacterial pathogens. *PLoS Pathogens*.

[9] Kováčová E., Kazár J., Šimková A. (1998). Clinical and serological analysis of a Q fever outbreak in western Slovakia with four-year follow-up. *European Journal of Clinical Microbiology and Infectious Diseases*.

[10] Paul S., Agger J. F., Markussen B., Christoffersen A.-B., Agerholm J. S. (2012). Factors associated with *Coxiella burnetii* antibody positivity in Danish dairy cows. *Preventive Veterinary Medicine*.

[11] Rousset E., Duquesne V., Russo P., Aubert M. F. (2010). Q fever. *Manual of Diagnostic Tests and Vaccines for Terrestrial Animals*.

[12] Haider N., Rahman M. S., Khan S. U. (2015). Serological evidence of *Coxiella burnetii* infection in cattle and goats in Bangladesh. *EcoHealth*.

[13] Talukder M. A. S., Khandoker M. A. M. Y., Rahman M. G. M., Islam M. R., Khan M. A. A. (2005). Reproductive problems of cow at Bangladesh Agricultural University Dairy Farm and possible remedies. *Pakistan Journal of Biological Sciences*.

[14] Khair A., Alam M. M., Rahman A. K. M. A., Islam M. T., Azim A., Chowdhury E. H. (2014). Incidence of reproductive and production diseases of cross-bred dairy cattle in Bangladesh. *Bangladesh Journal of Veterinary Medicine*.

[15] Sarker M. A. S., Aktaruzzaman M., Rahman A. K. M. A., Rahman M. S. (2014). Retrospective study of clinical diseases and disorders of cattle in Sirajganj district in Bangladesh. *Bangladesh Journal of Veterinary Medicine*.

[16] Rahman A. K. M. A., Saegerman C., Berkvens D. (2013). Bayesian estimation of true prevalence, sensitivity and specificity of indirect ELISA, Rose Bengal Test and Slow Agglutination Test for the diagnosis of brucellosis in sheep and goats in Bangladesh. *Preventive Veterinary Medicine*.

[17] Boarbi S., Mori M., Rousset E., Sidi-Boumedine K., Van Esbroeck M., Fretin D. (2014). Prevalence and molecular typing of *Coxiella burnetii* in bulk tank milk in Belgian dairy goats, 2009–2013. *Veterinary Microbiology*.

[18] Agger J. F., Christoffersen A.-B., Rattenborg E., Nielsen J., Agerholm J. S. (2010). Prevalence of *Coxiella burnetii* antibodies in Danish dairy herds. *Acta Veterinaria Scandinavica*.

[19] Muskens J., Van Engelen E., Van Maanen C., Bartels C., Lam T. J. G. M. (2011). Prevalence of *Coxiella burnetii* infection in Dutch dairy herds based on testing bulk tank milk and individual samples by PCR and ELISA. *Veterinary Record*.

[20] Czaplicki G., Houtain J.-Y., Mullender C. (2012). Apparent prevalence of antibodies to *Coxiella burnetii* (Q fever) in bulk tank milk from dairy herds in southern Belgium. *The Veterinary Journal*.

[21] Astobiza I., Ruiz-Fons F., Piñero A., Barandika J. F., Hurtado A., García-Pérez A. L. (2012). Estimation of *Coxiella burnetii* prevalence in dairy cattle in intensive systems by serological and molecular analyses of bulk-tank milk samples. *Journal of Dairy Science*.

[22] Lang G. H. (1989). Q fever: an emerging public health concern in Canada. *Canadian Journal of Veterinary Research*.

[23] Capuano F., Landolfi M. C., Monetti D. M. (2001). Influence of three types of farm management on the seroprevalence of Q fever as assessed by an indirect immunofluorescence assay. *Veterinary Record*.

[24] Ryan E. D., Kirby M., Collins D. M., Sayers R., Mee J. F., Clegg T. (2011). Prevalence of *Coxiella burnetii* (Q fever) antibodies in bovine serum and bulk-milk samples. *Epidemiology and Infection*.

[25] Khalili M., Sakhaee E. (2009). An update on a serologic survey of Q fever in domestic animals in Iran. *The American Journal of Tropical Medicine and Hygiene*.

[26] Ruiz-Fons F., Astobiza I., Barandika J. F. (2010). Seroepidemiological study of Q fever in domestic ruminants in semi-extensive grazing systems. *BMC Veterinary Research*.

[27] García-Pérez A. L., Astobiza I., Barandika J. F., Atxaerandio R., Hurtado A., Juste R. A. (2009). Short communication: investigation of *Coxiella burnetii* occurrence in dairy sheep flocks by bulk-tank milk analysis and antibody level determination. *Journal of Dairy Science*.

[28] Kennerman E., Rousset E., Gölcü E., Dufour P. (2010). Seroprevalence of Q fever (coxiellosis) in sheep from the Southern Marmara region, Turkey. *Comparative Immunology, Microbiology and Infectious Diseases*.

[29] Esmaeili S., Amiri F. B., Mostafavi E. (2014). Seroprevalence survey of Q fever among sheep in northwestern Iran. *Vector-Borne and Zoonotic Diseases*.

[30] Berri M., Souriau A., Crosby M., Rodolakis A. (2002). Shedding of *Coxiella burnetii* in ewes in two pregnancies following an episode of *Coxiella* abortion in a sheep flock. *Veterinary Microbiology*.

[31] Polydorou K. (1981). Q fever in Cyprus: a short review. *The British Veterinary Journal*.

